# GSH-Occ: Gradient-Shielded and Height-Aware BEV Occupancy Network

**DOI:** 10.3390/s26092800

**Published:** 2026-04-30

**Authors:** Bokai Ou, Tianhui Li, Zhigui Lin, Boao Wu, Pintong Chen, Zhajiacuo Zhou, Yating Liu, Jingyao Wang, Jinghua Guo, Lei He

**Affiliations:** 1 Institute of Artificial Intelligence, Xiamen University, Xiamen 361005, China; 36920241153243@stu.xmu.edu.cn; 2 SAIC GM Wuling Automobile Company Co., Ltd., Liuzhou 545007, China; tianhui.li@sgmw.com.cn (T.L.); zhigui.lin@sgmw.com.cn (Z.L.); 3 Guangxi Laboratory of New Energy Automobile, Liuzhou 545616, China; 4 Guangxi Key Laboratory of Automobile Four New Features, Liuzhou 545616, China; 5 School of Aerospace Engineering, Xiamen University, Xiamen 361102, China; 34520251151642@stu.xmu.edu.cn (B.W.); 34520251151621@stu.xmu.edu.cn (Z.Z.); 34520251151600@stu.xmu.edu.cn (Y.L.); wangjingyao1@xmu.edu.cn (J.W.); 6 Pen-Tung Sah Institute of Micro-Nano Science and Technology, Xiamen University, Xiamen 361005, China; 33520251153350@stu.xmu.edu.cn (P.C.); guojh@xmu.edu.cn (J.G.); 7 School of Vehicle and Mobility, Tsinghua University, Beijing 100084, China

**Keywords:** 3D occupancy prediction, BEV perception, residual learning, dual attention, height-aware voxelization

## Abstract

Camera-based 3D occupancy prediction commonly relies on bird’s-eye-view (BEV) representations, yet two limitations remain: optimization instability when inserting new modules into pre-trained BEV encoders, and height-agnostic BEV-to-voxel lifting that fails to preserve elevation-aware scene structure. We propose **GSH-Occ** (Gradient-Shielded and Height-Aware BEV Occupancy Network), a framework that tackles both issues through two complementary mechanisms. **Gradient-Shielded Residual Dual Attention (GS-RDA)** introduces a zero-initialized residual gate that preserves the identity mapping at initialization, allowing new attention modules to be grafted onto pre-trained encoders without disturbing learned features. **Height-Aware Adaptive Lift (HAL)** replaces naive channel replication with per-voxel adaptive fusion of BEV features and learnable height embeddings, followed by 3D convolutional refinement to capture vertical structure. On the Occ3D-nuScenes validation benchmark, GSH-Occ achieves 46.92 mIoU, outperforming FlashOcc by +3.40 mIoU. Ablation studies confirm that GS-RDA and HAL target distinct failure modes and yield complementary improvements.

## 1. Introduction

Bird’s-eye-view (BEV) representations have become the dominant paradigm for camera-based autonomous driving perception owing to their compact spatial layout and direct compatibility with downstream planning and control [1,2,3]. A common pipeline first transforms multi-view image features into a BEV feature map (e.g., via explicit depth discretization or learned view transformers [2,4,5]), then applies a task-specific head for detection, segmentation, or dense voxel prediction. For 3D occupancy prediction, casting the problem as a BEV task and then expanding vertically has proved to be a practical balance between accuracy and runtime cost [6,7]. In contrast, methods that operate directly in 3D volumes emphasize geometric fidelity but incur higher computational costs [8,9,10,11,12].

Despite these successes, two practical limitations persist. First, many BEV encoders recover height through simple channel-axis expansion, implicitly assuming uniform semantic distributions across elevation layers; this ignores layer-specific cues, such as ground surface, moving objects, and free space, and weakens vertical fidelity. Second, LSS-style view-transformer BEV features degrade at far ranges and in sparsely observed regions, yet most pipelines apply no recalibration before voxelization to compensate for these errors [5,7].

A further, orthogonal challenge arises during development and adaptation of large pre-trained BEV backbones: safely inserting new modules into trained networks. Randomly initialized attention blocks grafted onto a converged BEV encoder introduce high-variance gradients that disrupt the learned feature distribution, leading to loss spikes or sustained instability early in training. Existing workarounds—staged training, aggressive learning-rate warm-up, or partial freezing—are ad hoc and do not eliminate the underlying problem [13,14]. Techniques from adjacent domains have been proposed to address related problems: zero-initialized residual branches (ReZero) [15] improve gradient flow in deep networks, and adapter-style modules [16,17] enable parameter-efficient adaptation; neither, however, target the safe insertion of new components into feature-sensitive BEV encoders.

To address both the geometric shortfall of naive height expansion and the stable-integration problem, we propose **GSH-Occ** (Gradient-Shielded and Height-Aware BEV Occupancy Network), a structured approach to inserting modules into BEV occupancy pipelines. GSH-Occ rests on two complementary components: (1) **Gradient-Shielded Residual Dual Attention (GS-RDA)**, a dual-attention recalibration operator (channel and spatial) applied on the BEV plane and gated by a learnable scalar α initialized to zero. At initialization the block is an exact identity mapping; during training, α is optimized to smoothly activate the attention pathway, protecting the pre-trained feature distribution from initialization-induced disruption. (2) **Height-Aware Adaptive Lift (HAL)**, a voxelization module that substitutes simple channel replication with per-voxel fusion of BEV features and learnable height embeddings; a subsequent 3D convolutional stage then enforces vertical continuity and fills in elevation priors where image evidence is sparse.

Experiments on the nuScenes-Occ benchmark show that GSH-Occ achieves an improvement of +3.40 mIoU over a strong FlashOcc-based baseline.

**Figure 1:**
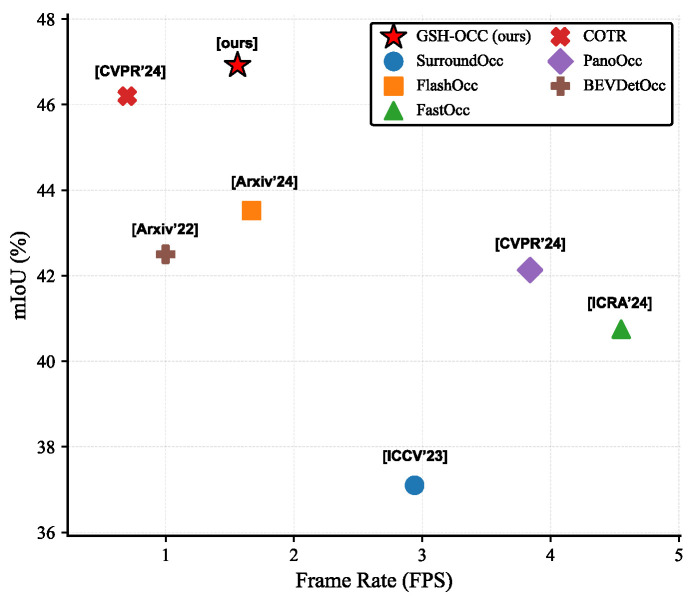
Accuracy–efficiency trade-off on the Occ3D-nuScenes validation set. The vertical axis reports mIoU (%) and the horizontal axis reports inference throughput (FPS). GSH-Occ achieves 46.92 mIoU at 1.56 FPS, the highest accuracy among all compared methods. COTR [18] attains 46.2 mIoU at a lower throughput of 0.70 FPS; FlashOcc [6] reaches 43.52 mIoU at 1.67 FPS. GSH-Occ gains +3.40 mIoU over FlashOcc at a cost of only −0.11 FPS, supporting the efficiency of the proposed components.

Figure 1 summarizes the accuracy–efficiency trade-off of representative camera-based occupancy methods on nuScenes-Occ. GSH-Occ achieves the highest mIoU (46.92%) among the compared methods while maintaining a frame rate of 1.56 FPS, showing that GS-RDA and HAL add little computational cost relative to the accuracy gains they bring.

Our contributions are threefold:

1. We identify the problem of safely inserting randomly initialized modules into pre-trained BEV encoders, characterize the problem’s gradient-level behavior through Jacobian analysis, and propose a principled solution (Section 2.3).
2. We propose GSH-Occ, instantiated through GS-RDA and HAL: GS-RDA employs a zero-initialized gate to ensure identity-preserving initialization and gradually activates dual-attention recalibration; HAL delivers height-aware voxelization through learnable elevation embeddings and per-voxel adaptive fusion (Section 2.4 and Section 2.5).
3. We empirically validate GSH-Occ on nuScenes-Occ, where the full model achieves 46.92 mIoU (+3.40 over baseline) with consistent improvements across semantic categories (Section 3).

## 2. Method

### 2.1. Problem Formulation

Let $I_{1:T}={\left\{{\left\{I_{i,t}\right\}}_{i=1}^{N_{c}}\right\}}_{t=1}^{T}$ denote $N_{c}$ calibrated surround-view images across *T* timesteps, with $I_{i,t}\in R^{3\times H_{I}\times W_{I}}$. 3D occupancy prediction requires learning a mapping

(1) $$\Phi :\;I_{1:T}\;⟶\;O\in {\left\{0,\ldots ,K-1\right\}}^{X\times Y\times Z},$$

where $O_{xyz}$ is the semantic label of voxel (x,y,z) and K=18 on Occ3D-nuScenes. The physical grid covers [−40,40]×[−40,40]×[−1,5.4] m^3^ discretized into a 200×200×16 lattice.

We decompose Φ into four sequential operators:

(2) Φ=H∘L∘R∘E,

where:

- $E:I_{1:T}\to F_{\mathrm{BEV}}\in R^{C\times H\times W}$ is the image encoder with LSS-style view transformation and temporal stereo fusion (C=256, H=W=200);
- $R:R^{C\times H\times W}\to R^{C\times H\times W}$ is the GS-RDA recalibration operator (Section 2.4);
- $L:R^{C\times H\times W}\to R^{C^{\prime }\times Z\times H\times W}$ is the HAL voxelization operator ($C^{\prime }=128$, Z=16; Section 2.5);
- $H:R^{C^{\prime }\times Z\times H\times W}\to R^{K\times X\times Y\times Z}$ is the occupancy classification head.

R and L are the two novel components; E and H are adopted from FlashOcc unchanged [6].

### 2.2. Overall Framework

**Figure 2:**
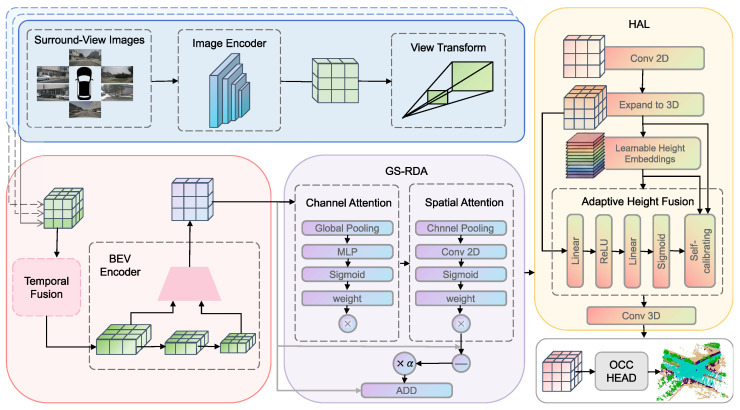
Overview of the proposed framework. **(i) Image encoding and view transformation:** A shared backbone encodes the surround-view images; an LSS-based stereo view transformer with depth supervision lifts the features into BEV space. **(ii) Temporal fusion and BEV encoding:** Multi-frame BEV features are ego-motion aligned, temporally fused, and encoded into a compact BEV representation. **(iii) GS-RDA:** Channel and spatial recalibration are applied in sequence; a learnable scalar α gates the residual branch, preventing disruption of pre-trained features at the start of training. **(iv) HAL:** The BEV feature is first processed by a 2D convolutional layer, then replicated along the vertical axis and combined with learnable height embeddings; a per-voxel sigmoid gate $\alpha_{xyz}$ blends the BEV evidence and the elevation prior as a convex combination, with the mixing ratio determined by the data. The resulting 3D voxel features are refined by a 3D convolutional layer and decoded by the occupancy head into per-voxel semantic predictions.

Figure 2 shows the architecture of GSH-Occ. Multi-view images are first processed by a shared backbone; the resulting multi-scale features are lifted into a BEV representation via view transformation and temporal stereo fusion. GS-RDA then recalibrates the BEV features along the channel and spatial dimensions through a zero-initialized gate, keeping training stable. HAL converts the refined BEV map into a 3D voxel volume by fusing BEV evidence with learnable height embeddings, improving vertical modeling. Finally, an occupancy head produces per-voxel semantic predictions.

### 2.3. Gradient-Shielded Residual Learning

Let ϕ denote the parameters of the pre-trained BEV encoder E, and let θ denote the parameters of a newly inserted module F(·;θ) initialized from $\theta ∼N(0,\sigma^{2}I)$. Define the *feature deviation* at position (i,j) as $\delta_{ij}=F{\left(x;\theta \right)}_{ij}-x_{ij}$. For a randomly initialized θ, $E[\delta_{ij}]=0$ but $\mathrm{Var}\left[\delta_{ij}\right]=O\left(\sigma^{2}C\right)$, which is O(1) for standard initializations with C=256. Left unchecked, these deviations reach the downstream head before ϕ has been updated at all.

Formulation

We cast module integration as a *residual interpolation*:

(3) $$y=x+\alpha \;\left(F(x;\theta )-x\right)=\left(1-\alpha \right)\;x+\alpha \;F\left(x;\theta \right),\;\alpha \in R,\;\alpha_{0}=0.$$

Identity Preservation

When α=0, y=x exactly, irrespective of the realization of θ. The outputs of E are therefore identical to those without the inserted module at the start of training, and no structural change to the base pipeline is needed.

Jacobian Analysis

The Jacobian of the output with respect to the BEV feature is:

(4) $$J_{x}^{y}=\frac{\partial y}{\partial x}=I+\alpha \;\frac{\partial F(x;\theta )}{\partial x}.$$

At α=0, $J_{x}^{y}=I$, so the Lipschitz constant of the residual block equals exactly 1. Gradients thus reach the pre-trained encoder E unscaled by the new module.

The gradient of the loss L with respect to the *encoder parameters* ϕ is:

(5) $$\frac{\partial L}{\partial \phi }=\frac{\partial L}{\partial y}\cdot \left[\left(1-\alpha \right)\;I+\alpha \;\frac{\partial F}{\partial x}\right]\cdot \frac{\partial x}{\partial \phi }.$$

At α=0, the bracketed term reduces to I, so the encoder receives gradients *identical* to those it would receive without the inserted module. As α grows, the term α ∂F/∂x introduces curvature from the new module, but by then θ has already been updated and F is no longer random.

The gradient with respect to the *module parameters* θ is:

(6) $$\frac{\partial L}{\partial \theta }=\alpha \cdot \frac{\partial L}{\partial y}\cdot \frac{\partial F(x;\theta )}{\partial \theta }.$$

The factor α acts as a self-regulating gate: at t=0, the module receives no gradient; as α is learned through backpropagation, the influence of F grows gradually without any hand-tuned warm-up schedule. Specifically, the update direction of α itself is:

(7) $$\frac{\partial L}{\partial \alpha }=\frac{\partial L}{\partial y}\cdot \left(F(x;\theta )-x\right).$$

In early training, when θ is random, F(x;θ)−x has zero mean and the gradient signal for α is weak. As θ converges, the deviation becomes task-aligned, pushing α toward positive values and gradually activating the attention branch.

Distinction from ReZero

ReZero [15] scales the *full branch output* F(x) by a scalar to improve gradient flow in deep homogeneous networks. Gradient-Shielded Residual Learning (GSRL) instead gates the *deviation* F(x)−x, so that y=x exactly when α=0 regardless of network topology or whether an external skip connection exists. More fundamentally, GSRL is designed to suppress output perturbation when a new module is inserted into a feature-sensitive intermediate representation—a goal distinct from ReZero’s focus on gradient propagation depth at initialization.

### 2.4. Gradient-Shielded Residual Dual Attention

We instantiate F(·) in Equation (3) as a sequential dual-attention module [19] applied to BEV feature maps.

#### 2.4.1. Channel Attention

Given $x\in R^{C\times H\times W}$, global average and max pooling produce compact channel statistics:

(8) $$z_{\mathrm{avg}}=\mathrm{GAP}\left(x\right),\;z_{\max}=\mathrm{GMP}\left(x\right)\;\in \;R^{C}.$$

A shared two-layer MLP with bottleneck ratio r=16,

(9) $$\mathrm{MLP}\left(z\right)=W_{2}\;\sigma_{\mathrm{ReLU}}\left(W_{1}\;z\right),\;W_{1}\in R^{C/r\times C},\;W_{2}\in R^{C\times C/r},$$

processes both descriptors; the outputs are summed and passed through a sigmoid:

(10) $$w_{c}=\sigma \left(\mathrm{MLP}\left(z_{\mathrm{avg}}\right)+\mathrm{MLP}\left(z_{\max}\right)\right)\in {\left(0,1\right)}^{C}.$$

The channel-recalibrated feature is $x^{\prime }=w_{c}⊙x$, where ⊙ denotes channel-wise multiplication.

#### 2.4.2. Spatial Attention

Channel-wise average and max pooling of $x^{\prime }$ yield two spatial maps, which are concatenated and processed by a 7×7 convolution:

(11) $$w_{s}=\sigma \;\left({\mathrm{Conv}}_{7\times 7}\left([{\mathrm{Avg}}_{c}\left(x^{\prime }\right);\;{\mathrm{Max}}_{c}\left(x^{\prime }\right)]\right)\right)\in {\left(0,1\right)}^{H\times W}.$$

The dual-attention output is:

(12) $$F_{\mathrm{DA}}\left(x\right)=w_{s}⊙x^{\prime }.$$

The full GS-RDA operator follows by setting $F=F_{\mathrm{DA}}$ in Equation (3).

### 2.5. Height-Aware Adaptive Lift

#### 2.5.1. Motivation

Existing occupancy heads based on BEV pipelines [6,7] project the 2D BEV feature map to 3D through a simple channel-axis expansion:

(13) $$\hat{O}=\mathrm{Reshape}\left(\mathrm{Linear}(F_{\mathrm{BEV}})\right),$$

where the linear layer maps *C* channels to Z×K output units. This treats every height layer as the same linear combination of the BEV evidence, ignoring any prior knowledge of 3D scene structure. In practice, outdoor scenes follow clear elevation-dependent patterns: ground and road markings appear in the lowest layers (z≤0 m), moving objects fill the mid-range (z∈[0.5,2.5] m), and free space or tall structures occupy the upper layers. Equation (13) has no way to encode or exploit these patterns.

#### 2.5.2. Learnable Height Embeddings

We introduce a set of trainable height embedding vectors ${\left\{h_{z}\right\}}_{z=0}^{Z-1}$, $h_{z}\in R^{C}$, one per elevation layer, initialized by truncated normal sampling N(0,0.02) and updated by gradient descent throughout training. These embeddings let the network learn elevation-specific representations for occupancy classification and act as fallback priors where image evidence is weak.

#### 2.5.3. Adaptive Height Fusion

Let $f\left(x,y\right)\in R^{C}$ be the BEV feature at location (x,y) after 2D convolutional pre-processing. For each voxel (x,y,z), the BEV feature and the height embedding are concatenated:

(14) $$g_{xyz}=\left[f\left(x,y\right);\;h_{z}\right]\in R^{2C}.$$

A two-layer MLP maps $g_{xyz}$ to a channel-wise gate:

(15) $$\alpha_{xyz}=\sigma \left(W_{2}\;\sigma_{\mathrm{ReLU}}\left(W_{1}\;g_{xyz}\right)\right)\in {\left(0,1\right)}^{C},$$

where $W_{1}\in R^{C\times 2C}$ and $W_{2}\in R^{C\times C}$ are initialized with He initialization [20]. The lifted feature is the element-wise convex combination:

(16) $$f_{\mathrm{lift}}\left(x,y,z\right)=\alpha_{xyz}⊙f\left(x,y\right)+\left(1-\alpha_{xyz}\right)⊙h_{z}.$$

The convex combination bounds the variance of the fused representation, avoiding the instability of unbounded multiplicative interactions and is better conditioned than additive or purely multiplicative alternatives.

##### Self-Calibrating Behavior

The gate $\alpha_{xyz}$ depends on both the local BEV feature f(x,y) and the elevation index *z* (through $h_{z}$) and has two interpretable limiting cases. When BEV evidence is reliable (nearby objects with dense multi-view overlap), the gate saturates toward α→1, so Equation (16) reduces to $f_{\mathrm{lift}}\approx f\left(x,y\right)$, matching the naive BEV-copy baseline. When evidence is unreliable (far-range or occluded regions where depth ambiguity is large and BEV features degrade), α→0 and the output reverts to the learned elevation prior $h_{z}$. This behavior matches the depth uncertainty of monocular-rig cameras, where range error grows as $\sigma_{d}^{2}\propto d^{2}$ [5], and needs no explicit range-dependent supervision.

HAL also subsumes naive lifting as a special case: setting $\alpha_{xyz}\equiv 1$ and absorbing the linear map of Equation (13) into the pre-processing convolution recovers the baseline exactly.

##### Relationship to GSRL

Equations (3) and (16) share the convex-combination form but serve different purposes. In GSRL, α is a *scalar initialized to zero* whose role is to suppress output-space perturbation at module insertion time. In HAL, $\alpha_{xyz}$ is a *spatial- and channel-varying* function of the current input, computed on the fly to select between observed BEV features and learned elevation priors. No gradient shielding is needed in HAL because it replaces an existing module rather than augmenting it and therefore does not risk disrupting pre-trained features.

#### 2.5.4. 3D Convolutional Refinement

The fused volume $F_{\mathrm{lift}}\in R^{C\times Z\times H\times W}$ is passed through a 3×3×3 convolutional layer:

(17) $$F_{\mathrm{refined}}=\mathrm{BN}\left(\mathrm{Conv}3D_{k=3}\left(F_{\mathrm{lift}}\right)\right).$$

Unlike 2D operations that process each height slice independently, this layer aggregates information across adjacent elevation layers, promoting continuity for objects spanning multiple heights (e.g., vehicles from z=0.5 m to z=2.5 m) and implicitly regularizing the height embeddings $h_{z}$.

## 3. Experiments

This section describes the dataset, implementation details, and evaluation metric used in all experiments.

Dataset

We evaluate on nuScenes-Occ [8], derived from nuScenes [21]. Voxel-level semantic labels are provided on a 200×200×16 grid covering [−40,40] m in x/y and [−1,5.4] m in *z*, with K=18 semantic classes. We use the standard split of 700 training and 150 validation scenes.

Implementation

GSH-Occ is built on the FlashOcc pipeline with a Swin-Transformer-Base backbone pre-trained on ImageNet-22K [22], 512×1408 input resolution, and four-frame temporal stereo fusion. The BEV encoder outputs a 200×200 feature map with C=256 channels. GS-RDA (r=16, 7×7 spatial kernel) is inserted after the BEV encoder neck; HAL operates inside the occupancy head with Z=16 and C=128.

All models are trained for 24 epochs using AdamW [23] with base learning rate $2\times 10^{-4}$, weight decay $10^{-2}$, and a step decay schedule with the learning rate decayed by a factor of 10 at epoch 24 on four NVIDIA RTX 3090 GPUs (NVIDIA Corporation, Santa Clara, CA, USA; batch size 2 per GPU). All experiments were implemented in PyTorch 1.10.0 (Meta AI; https://pytorch.org, accessed on 24 April 2026) with CUDA 11.1, using MMCV 1.5.3, MMDetection 2.25.1, and MMSegmentation 0.25.0 from the OpenMMLab framework (https://github.com/open-mmlab, accessed on 24 April 2026).

Metrics

We report mIoU averaged over K=18 semantic classes under the Occ3D evaluation protocol [8]. For class *c*:

(18) $${\mathrm{IoU}}_{c}=\frac{|{\hat{V}}_{c}\cap V_{c}|}{|{\hat{V}}_{c}\cup V_{c}|},\;\mathrm{mIoU}=\frac{1}{K}\sum_{c=1}^{K}{\mathrm{IoU}}_{c},$$

where ${\hat{V}}_{c}$ and $V_{c}$ denote predicted and ground-truth occupied voxel sets for class *c*. Evaluation covers only camera-visible voxels, excluding occluded regions where annotations are sparse, following [6,8].

## 4. Results

We report quantitative comparisons, qualitative visualizations, and ablation experiments on the nuScenes-Occ validation set under the setup described in Section 3.

### 4.1. Comparison with State of the Art

Table 1 compares GSH-Occ with published baselines on the nuScenes-Occ validation set. GSH-Occ achieves 46.92 mIoU, the highest among all compared methods. Notably, OCCFusion [24] (46.79) relies on camera–LiDAR fusion, while GSH-Occ attains superior accuracy with camera input alone. COTR [18] reaches 46.2 mIoU with camera only, at a substantially lower throughput of 0.70 FPS versus our 1.56 FPS. No changes are made to the training schedule, loss function, or data augmentation.

Figure 1 shows the accuracy–efficiency trade-off: GSH-Occ gains +3.40 mIoU over FlashOcc at a cost of only 0.11 FPS (1.67 → 1.56 FPS).

### 4.2. Qualitative Analysis

**Figure 3:**
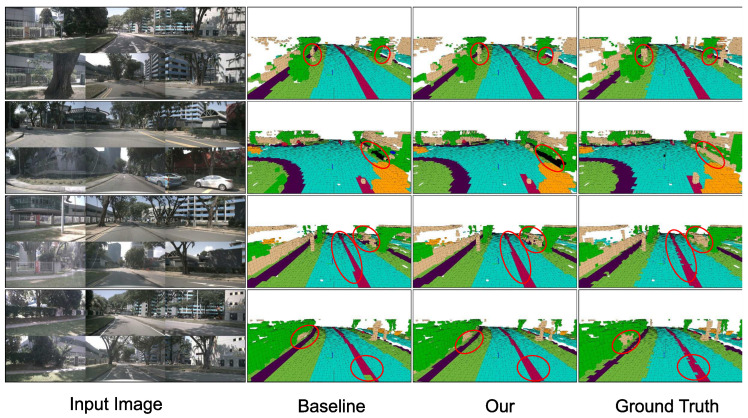
Qualitative comparison on daytime scenes from nuScenes-Occ. Each row corresponds to an independent validation scene. Columns (left to right): (1) surround-view input images (front-left, front, front-right in the upper sub-row; rear-left, rear, rear-right in the lower sub-row); (2) FlashOcc baseline prediction; (3) GSH-Occ (ours); (4) ground-truth semantic occupancy. Red circles highlight regions discussed in the text.

**Figure 4:**
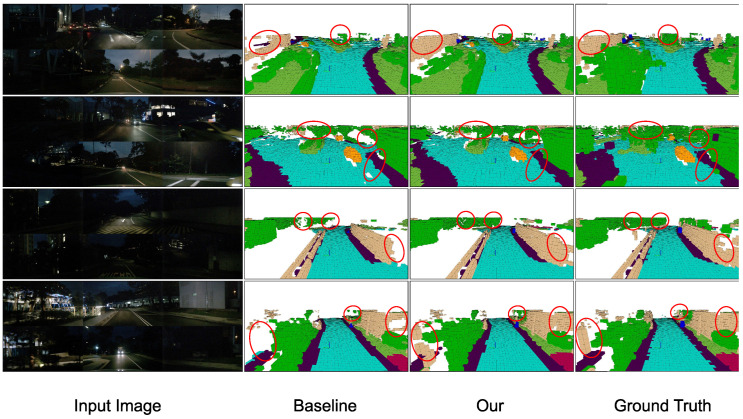
Qualitative comparison on nighttime scenes from nuScenes-Occ. Each row corresponds to an independent nighttime validation scene. Column layout follows Figure 3. Low illumination degrades raw image quality and reduces depth estimation reliability, exposing additional failure modes of the baseline that GSH-Occ mitigates through BEV recalibration and height-aware voxelization. Red circles highlight regions discussed in the text.

Figure 3 and Figure 4 compare FlashOcc and GSH-Occ on eight validation scenes from nuScenes-Occ: four daytime scenes (Figure 3) and four nighttime scenes under low illumination (Figure 4). Each row shows, left to right: the input image, the baseline prediction, the GSH-Occ prediction, and the ground-truth occupancy volume rendered from the same viewpoint.

Three consistent patterns emerge across both daytime and nighttime conditions.

**(i) Large static structures.** The baseline produces fragmented predictions and isolated misclassifications near building facades and vegetation boundaries. GSH-Occ suppresses these inconsistencies through the GS-RDA recalibration branch and the 3D convolutional refinement in HAL (Equation (17)), yielding coherent boundaries across adjacent elevation layers. Under nighttime conditions (Figure 4), where image contrast is low, the baseline further breaks down at distant structure boundaries; GS-RDA spatial recalibration down-weights spatially inconsistent activations before voxelization, recovering more coherent predictions.

**(ii) Occluded and far-range regions.** In occluded and far-range areas, the baseline generates false-positive free-space voxels and discontinuous predictions. GSH-Occ falls back to the learned elevation prior $h_{z}$ (Equation (16)) where BEV evidence is weak, producing predictions that better conform to the actual scene layout. At night, degraded depth estimates amplify this effect; the HAL gate $\alpha_{xyz}$ shifts toward zero in such regions, and the height embeddings supply stable elevation priors that the baseline entirely lacks (Figure 4).

**(iii) Driveable surface and ground-level geometry.** The baseline introduces occasional fragmentation along driveable-surface boundaries, whereas GSH-Occ produces spatially continuous predictions. The ground-truth annotations themselves contain surface gaps in places, yet GSH-Occ recovers a more coherent ground plane in these regions. Quantitatively, GSH-Occ achieves an IoU of 86.25% on the driveable-surface category, compared with 83.87% for the baseline (+2.38 pp; Table 1).

Taken together, GS-RDA and HAL address complementary failure modes of the baseline, and their gains remain consistent across diverse illumination conditions. Residual errors in highly dynamic scenes with dense occlusions suggest that incorporating temporal modeling could further improve performance.

### 4.3. Ablation Study

Table 2 shows results for four variants trained under identical settings on nuScenes-Occ.

**GS-RDA:** Channel and spatial recalibration improve overall mIoU, indicating that the LSS encoder produces BEV features with unevenly distributed channel and spatial activations that attention can correct. This +1.19 gain requires no change to the voxelization pipeline, confirming that GS-RDA and HAL target independent aspects of the model.

**HAL:** Replacing naive linear expansion with adaptive height fusion yields a +0.92 gain concentrated in categories with strong vertical extent (vehicles, buildings, vegetation; Table 1), confirming that height embeddings supply information absent from 2D BEV features.

**Interaction:** The observed $\Delta_{\mathrm{joint}}=+3.40$ exceeds $\Delta_{R}+\Delta_{L}=+2.11$, suggesting that the two modules are complementary within Φ (Equation (2)). GS-RDA improves the quality of $F_{\mathrm{BEV}}$, which provides more stable input to the HAL gate $\alpha_{xyz}$ (Equation (15)) during BEV-to-voxel mixing. Meanwhile, the enhanced voxel predictions from HAL lead to more structured supervision for upstream BEV features. Overall, the combined gain slightly exceeds the sum of individual improvements (+1.29 mIoU), indicating a mild cooperative effect.

## 5. Discussion

Interpreting the headline result.

GSH-Occ achieves 46.92 mIoU on the nuScenes-Occ validation set, surpassing all camera-only baselines and narrowly exceeding OCCFusion (46.79) [24], which pairs cameras with LiDAR. LiDAR provides metric-scale range measurements that directly resolve the monocular depth ambiguity central to camera-only lifting; the fact that GSH-Occ closes—and marginally exceeds—this gap indicates that structural priors targeting feature imbalance and height ambiguity can largely substitute for hardware-level depth sensing in the occupancy task. Per-class results in Table 1 reinforce this reading: GSH-Occ leads on static scene categories (barrier: +7.55 pp and driveable surface: +7.46 pp over OCCFusion), where the absence of dense dynamic clutter makes structural priors particularly effective.

Synergy between GS-RDA and HAL.

The ablation study in Table 2 shows that the joint gain ($\Delta_{\mathrm{joint}}=+3.40$ mIoU) exceeds the sum of individual contributions ($\Delta_{R}+\Delta_{L}=+2.11$) by +1.29 mIoU. This super-additive effect has a mechanistic basis. GS-RDA recalibrates $F_{\mathrm{BEV}}$ before voxelization, producing features with more uniform channel activations and spatially consistent responses; the adaptive gate $\alpha_{xyz}$ in HAL (Equation (15)) then operates on this improved representation, yielding more reliable mixing weights and sharper elevation assignments. In the reverse direction, crisper per-voxel predictions from HAL deliver stronger categorical supervision that propagates upstream and further refines BEV features. The two modules thus address complementary failure modes and reinforce each other in a closed training loop.

Comparison with competing design choices.

COTR [18] reaches 46.2 mIoU through dense 3D cross-attention queries but at 0.70 FPS—less than half the throughput of GSH-Occ (1.56 FPS). GSH-Occ surpasses COTR on both metrics by augmenting an efficient two-stage pipeline rather than redesigning it. Targeted interventions at the BEV encoder and lifting stages suffice to outperform architecturally heavier alternatives. More broadly, the per-class pattern in Table 1 suggests that the primary bottleneck in camera-only occupancy prediction is not global representational capacity but rather feature imbalance and height ambiguity at specific points in the pipeline—both of which GSH-Occ addresses directly.

Practical implications.

The zero-initialized residual gate in GS-RDA guarantees that inserting the module into a pre-trained BEV encoder leaves the initial forward pass and all upstream gradients unchanged. GSH-Occ can therefore be initialized from an existing FlashOcc checkpoint and fine-tuned without staged training or learning-rate warm-up—a concrete advantage over approaches that require training from scratch. HAL’s gate defaults to identity-equivalent behavior ($\alpha_{xyz}\;\equiv \;1$) at initialization, so the lifting stage begins from a known-good solution and the new degrees of freedom are activated gradually through standard gradient descent. GSH-Occ thus integrates into an existing deployment pipeline through fine-tuning alone, with no structural changes to the surrounding architecture.

## 6. Conclusions

We presented GSH-Occ, a camera-based BEV occupancy framework that addresses two distinct limitations through GS-RDA and HAL. GS-RDA addresses the stable integration problem with a zero-initialized residual gate: the block Jacobian equals the identity at initialization, leaving encoder gradients unperturbed; α is then learned by standard backpropagation, gradually activating dual-attention recalibration without any manual scheduling. HAL replaces height-agnostic lifting with learnable elevation embeddings and per-voxel convex fusion, followed by 3D convolutional refinement; the gate degrades gracefully under depth uncertainty in a manner consistent with monocular range error, and the formulation reduces to naive channel expansion when $\alpha_{xyz}\equiv 1$. On nuScenes-Occ, GSH-Occ reaches 46.92 mIoU, a gain of +3.40 over the FlashOcc baseline.

Limitations and Future Directions

GS-RDA uses a single global scalar α; a spatially varying gate $\alpha \in R^{H\times W}$ could allow location-dependent activation at the cost of more parameters and potential overfitting. HAL’s per-voxel MLP captures inter-height context only through the 3×3×3 convolution; replacing this with vertical attention is a natural next step. Both components target camera-only pipelines; how they interact with LiDAR-based BEV encoders, where depth confidence is more spatially uniform, remains to be explored.

## Figures and Tables

**Table 1:** 3D occupancy prediction on the Occ3D-nuScenes validation set. Per-class IoU and mIoU are reported. **Bold**: best per column. C/L/R/S: camera/LiDAR/radar/satellite. ✓: visible-mask supervision used; –: not reported.

Method	Input	Visible Mask	mIoU	■ Others	■ Barrier	■ Bicycle	■ Bus	■ Car	■ Const. Veh.	■ Motorcycle	■ Pedestrian	■ Traffic Cone	■ Trailer	■ Truck	■ Drive. Suf.	■ Other Flat	■ Sidewalk	■ Terrain	■ Manmade	■ Vegetation
TPVFormer [12]	C	✓	34.2	7.68	44.01	17.66	40.88	46.98	15.06	20.54	24.69	24.66	24.26	29.28	79.27	40.65	48.49	49.44	32.63	29.82
SurroundOcc [9]	C	✓	37.1	8.97	46.33	17.08	46.54	52.01	20.05	21.47	23.52	18.67	31.51	37.56	81.91	41.64	50.76	53.93	42.91	37.16
OccFormer [11]	C	✓	37.4	9.15	45.84	18.20	42.80	50.27	24.00	20.80	22.86	20.98	31.94	38.13	80.13	38.24	50.83	54.30	46.41	40.15
VoxFormer [25]	C	✓	40.7	–	–	–	–	–	–	–	–	–	–	–	–	–	–	–	–	–
FBOcc [26]	C	✓	42.1	14.30	49.71	30.00	46.62	51.54	29.30	29.13	29.35	30.48	34.97	39.36	83.07	47.16	55.62	59.88	44.89	39.58
PanoOcc [27]	C	–	42.13	11.67	50.48	29.64	49.44	55.52	23.29	33.26	30.55	30.99	34.43	42.57	83.31	44.23	54.40	56.04	45.94	40.40
FastOcc [28]	C	✓	40.75	12.86	46.58	29.93	46.07	54.09	23.74	31.10	30.68	28.52	33.08	39.69	83.33	44.65	53.90	55.46	42.61	36.50
BEVDet4D [7]	C	✓	42.5	12.37	50.15	26.97	51.86	54.65	28.38	28.96	29.02	28.28	37.05	42.52	82.55	43.15	54.87	58.33	48.78	43.79
FlashOcc [6]	C	✓	43.52	13.31	51.62	28.07	50.91	55.69	27.46	31.05	29.98	29.20	38.86	43.68	83.87	45.63	56.33	59.01	50.63	44.56
GEOcc [29]	C	✓	44.67	14.02	51.40	33.08	52.08	56.72	30.04	33.54	32.34	35.83	39.34	44.18	83.49	46.77	55.72	58.94	48.85	43.00
DepthOcc-L [30]	C	✓	45.3	–	–	–	–	–	–	–	–	–	–	–	–	–	–	–	–	–
SA-Occ [31]	C+S	–	44.64	13.70	51.10	30.40	52.40	56.90	27.00	31.60	30.20	30.30	37.60	44.00	84.80	46.10	58.60	62.10	53.10	49.10
HyDRa [32]	C+R	–	44.40	–	–	–	52.30	56.30	–	35.90	35.10	–	–	44.10	–	–	–	–	–	–
COTR [18]	C	✓	46.2	**14.85**	53.25	**35.19**	50.83	57.25	**35.36**	34.06	33.54	**37.14**	38.99	44.97	84.46	48.73	57.60	61.08	51.61	46.72
OCCFusion [24]	C+L	–	46.79	11.65	47.81	32.07	**57.27**	57.51	31.80	**40.11**	**47.35**	33.74	**45.81**	**50.35**	78.79	37.17	44.36	53.36	**63.18**	**63.20**
**GSH-Occ (Ours)**	C	✓	**46.92**	14.81	**55.36**	29.15	53.83	**59.47**	31.47	33.12	33.19	34.16	37.70	45.21	**86.25**	**51.25**	**60.06**	**64.36**	57.90	50.38

**Table 2:** **Module-level ablation of GSH-Occ.** All variants share the same training configuration. Δ: gain over baseline. **Bold**: best result.

Configuration	mIoU	Δ
Baseline (FlashOcc)	43.52	–
+GS-RDA	44.71	+1.19
+HAL	44.44	+0.92
+GS-RDA + HAL (GSH-Occ)	**46.92**	+**3.40**

## Data Availability

The experiments in this study were conducted on the nuScenes dataset, which is publicly available at https://www.nuscenes.org/ (accessed on 22 April 2026). The occupancy annotations used for the nuScenes-Occ benchmark are publicly available at https://github.com/CVPR2023-3D-Occupancy-Prediction/CVPR2023-3D-Occupancy-Prediction (accessed on 22 April 2026). No new datasets were created in this study.

## Abbreviations

The following abbreviations are used in this manuscript:

BEV	Bird’s-Eye View
GSH-Occ	Gradient-Shielded and Height-Aware BEV Occupancy Network
GS-RDA	Gradient-Shielded Residual Dual Attention
GSRL	Gradient-Shielded Residual Learning
HAL	Height-Aware Adaptive Lift
LSS	Lift-Splat-Shoot

mIoU	Mean Intersection over Union
IoU	Intersection over Union
FPS	Frames Per Second
MLP	Multi-Layer Perceptron
GAP	Global Average Pooling
GMP	Global Max Pooling
CBAM	Convolutional Block Attention Module
